# Prediction of Antimicrobial Peptides Based on Sequence Alignment and Support Vector Machine-Pairwise Algorithm Utilizing LZ-Complexity

**DOI:** 10.1155/2015/212715

**Published:** 2015-02-23

**Authors:** Xin Yi Ng, Bakhtiar Affendi Rosdi, Shahriza Shahrudin

**Affiliations:** ^1^School of Electrical & Electronic Engineering, Universiti Sains Malaysia, 14300 Nibong Tebal, Seberang Perai Selatan, Pulau Pinang, Malaysia; ^2^Intelligent Biometric Group, School of Electrical & Electronic Engineering, USM Engineering Campus, Universiti Sains Malaysia, 14300 Nibong Tebal, Seberang Perai Selatan, Pulau Pinang, Malaysia; ^3^School of Pharmacy, Universiti Sains Malaysia, 11800 Minden, Pulau Pinang, Malaysia

## Abstract

This study concerns an attempt to establish a new method for predicting antimicrobial peptides (AMPs) which are important to the immune system. Recently, researchers are interested in designing alternative drugs based on AMPs because they have found that a large number of bacterial strains have become resistant to available antibiotics. However, researchers have encountered obstacles in the AMPs designing process as experiments to extract AMPs from protein sequences are costly and require a long set-up time. Therefore, a computational tool for AMPs prediction is needed to resolve this problem. In this study, an integrated algorithm is newly introduced to predict AMPs by integrating sequence alignment and support vector machine- (SVM-) LZ complexity pairwise algorithm. It was observed that, when all sequences in the training set are used, the sensitivity of the proposed algorithm is 95.28% in jackknife test and 87.59% in independent test, while the sensitivity obtained for jackknife test and independent test is 88.74% and 78.70%, respectively, when only the sequences that has less than 70% similarity are used. Applying the proposed algorithm may allow researchers to effectively predict AMPs from unknown protein peptide sequences with higher sensitivity.

## 1. Introduction

Recently, antimicrobial peptides (AMPs) have been used in drug design to fight many types of microorganisms such as bacteria, fungi, parasites, enveloped viruses, and cancer cells [1]. AMPs kill microorganisms through disruption of membrane integrity and are believed to be less likely to induce resistance [2]. It is believed that AMPs can substitute the traditional antibiotics as AMPs can be used to overcome the growing problems of antibiotic resistance [3].

AMPs are a group of molecules that form an important part of the innate immune system. Generally, AMPs consist of 12 to 100 amino acid residues and can be found among all classes of life including bacteria, fungi, plants, invertebrates, and vertebrates [3, 4]. Generally, by referring to their activities, structural properties, and sequence features, AMPs can be classified into several main categories such as antibacterial, antifungal, antiviral, antitumor and anticancer [5, 6].

Over the last few decades, as researchers and scientists are looking in new drugs and drugs targets, AMPs have been raised as new interest. Due to their short length and rapid and efficient effect against microbes, AMPs have become potential candidates as peptide drugs. There are some AMPs and their derivatives which have already passed the clinical trials successfully and some AMPs are being considered to become therapeutics [5]. However, the experimental identification and designing of the AMPs are expensive and also time and resources consuming [7]. Therefore, it is necessary to develop a high accuracy computational method which is able to predict these AMPs sequences effectively.

Most researchers are concentrating on discovering new in silico tools for antimicrobial peptide prediction as computational approaches can accelerate the process of antimicrobial drug discovery and design [8]. Many computational methods have been introduced to predict AMPs based on the different features of AMPs such as AntiBP method [1], CAMP methods [5], the combination of sequence alignment and the feature selection method [9], and the pseudo amino acid composition [10, 11].

The AntiBP method had been used to predict the antibacterial peptides. The N- and C-terminal residues are used for predicting antibacterial peptides using support vector machine (SVM), quantitative matrices (QM), and artificial neural network (ANN). Their training sets are limited to N and/or C terminal residue peptides. Unfortunately, the AMPs have much variation in size but these machine learning methods only work well at fixed lengths [1]. For the CAMP methods [5], the AMPs prediction is performed using random forests (RF), SVM, and discriminant analysis (DA) based on all classes of full AMPs sequences. The sequence alignment method [9] enjoys high prediction accuracy but it is not able to predict all sequences. This is because the classification concept used in the sequence alignment relies on HSPs scores which represent the similarity scores between two sequences using BLASTP. If the test sequence has no relationship with any training sequence, a HSPs score cannot be generated; thus the classification concept cannot be performed on that particular sequence.

To solve the problem of the sequence alignment, in [9], they utilize the concept of amino acid composition and pseudo amino acid composition (PseAAC) to represent the AMPs sequence. Then, the maximum relevance minimum redundancy (mRMR) method [12] and incremental feature selection (IFS) method [13, 14] are applied to select the optimal feature for prediction. Finally, the prediction was developed using the nearest neighbor algorithm (NNA) [15]. This method has lower performance accuracy than other methods. Similar to the method proposed in [9–11], the PseAAC is applied to represent the AMPs sequence. But, they do not utilize the feature selection method as what was performed in [9]. Instead, they improve the performance of the prediction by utilizing the Fuzzy K-nearest neighbor algorithm (F-kNN) [10] and support vector machine (SVM) [11]. The performance is improved by utilizing F-kNN and SVM as the classifiers. However, the problems of using PseAAC as the feature extraction technique still exist where the researchers might face difficulty determining the value of parameter, *λ*. In order to use the PseAAC for reflecting the input protein sequences, the value of parameter *λ* must be nonnegative integer and should not be larger than the length of input protein sequence [16]. Because the length of input sequences is varied and some of them are equal to 1, the value of the PseAAC parameter, *λ*, is difficult to determine for an optimal result of AMPs prediction.

In short, none of them has successfully identified which AMPs feature is the most suitable for accurately predicting AMPs. Therefore, a new computational method must be proposed to overcome those problems existing in previous predictors, as well as to predict AMPs accurately and effectively. This will hasten the discovery and design process of AMPs. Thus, in this study, a new algorithm for AMPs prediction is proposed by combining the sequence alignment method, Lempel-Ziv (LZ) complexity [17], and support vector machines- (SVMs-) pairwise algorithm [18, 19].

The concept of SVM-pairwise algorithm was introduced by [19] with the aim of detecting remote protein evolutionary and structural relationships. In [18, 19], BLAST is used to generate the pairwise similarity scores of each test sequence against all other sequences of the training set. In this paper, instead of using the BLAST, we use the LZ complexity algorithm [17] in the computation of the pairwise similarity scores. The new proposed concept of SVM-LZ complexity pairwise algorithm is the combination of LZ complexity and SVM classification. To the best of our knowledge, this concept has never been implemented for AMPs prediction and also for the other type of bioinformatics applications. By implementing the SVM-pairwise with LZ complexity algorithm into the proposed algorithm, all training sequences can be predicted without facing the parameter value selection problem of the feature selection method. This method provides a relatively high sensitivity performance for AMPs prediction compared to CAMP methods [5] and the integrated method proposed by Wang and colleagues [9].

## 2. Materials and Methods

### 2.1. Datasets

*Training Sets.* There are two types of training set, which are “normal training set” and “<0.7 training set.” Both of the training sets were downloaded from the website provided by Wang et al. [9]. The normal training set consists of 2752 sequences in the positive training set and 10014 sequences in the negative training set. These training sequences were downloaded from CAMP [5] and processed by Wang et al. [9].

The <0.7 training set is the subset of the normal training set. It is known that the performance of AMPs predictor will be affected by homologous sequences in the datasets. Therefore, Wang et al. [9] have prepared a new training set by eliminating the homologous sequences inside the normal training set with a cutoff threshold of 70%. The homologous sequences which have equal to or greater than 70% sequence identity compared to other training and test sequences have been removed. After the elimination process, the <0.7 training set has 870 positive training sequences and 8861 negative training sequences.

In [9], the jackknife test and independent test were performed using two different training sets. The jackknife test was performed using the <0.7 training set while the normal training set was used to perform independent test. Unlike [9], in this study, jackknife test and independent test were performed on both <0.7 and normal training sets to clearly present the effectiveness of the proposed algorithm.

*Test Sets.* There are two types of dataset that have been used for the independent test, which are “Wang test set” and “CAMP test set.” The “Wang test set” was downloaded from Wang et al. [9] and, after eliminating those sequences with nonstandard residues, the set consists of 1136 AMPs sequences. This test set has been used to compare the performance of the proposed method with the method proposed by Wang et al. [9]. As for the “CAMP test set,” the sequences were downloaded from the new release of CAMP database [20]. The updated database contains 2438 AMPs sequences which are identified without experimental evidences. After eliminating those sequences containing nonstandard residues, this test set consists of 2420 AMPs sequences. This test set is used to confirm that our proposed method can be used for the recently identified peptide sequences.

### 2.2. Proposed Algorithm

The proposed algorithm for AMPs prediction in this study is divided into two main stages. The two main stages include the sequence alignment method [9] and support vector machines- (SVMs-) LZ complexity pairwise algorithm [17–19]. First, the sequence alignment method [9] is used to predict AMPs sequences. Then, the remaining sequences are predicted using SVM-LZ complexity pairwise algorithm, since the sequence alignment method cannot predict all peptide sequences [9].

#### 2.2.1. Sequence Alignment Method

**Figure 1:**
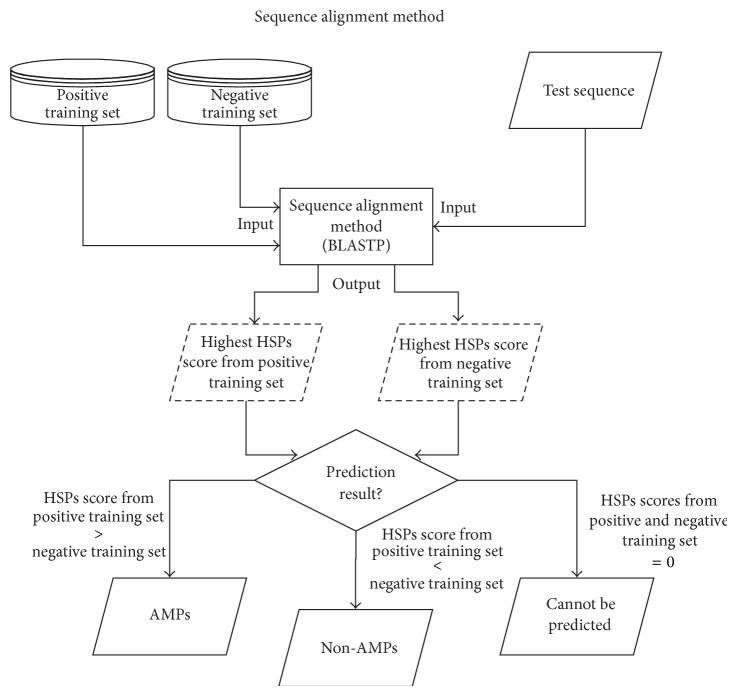
The flowchart of the sequence alignment method for stage 1.

The sequence alignment method [9] is suitable for predicting AMPs sequences as a peptides function is highly related to its sequential order. In this study, BLASTP [21] is used as sequence alignment method to predict AMPs.  Figure 1 shows a flow chart of the method.

The first step of the sequence alignment is the preparation of databases. In order to predict AMPs, two databases are needed to represent the sequences from both negative and positive training sets. The BLASTP is used to predict test sequences with default parameter based on the databases in this study. The high-scoring segment pairs (HSPs) scores are calculated by the BLASTP based on test sequence and all training sequences in both databases. These HSPs scores reflect the similarity between the test sequence and all training sequences in the training sets. Since the test sequence is compared to both databases, two highest HSPs scores could be obtained from both databases.

In the final step, both of the maximum HSPs scores are compared. If the HSPs score from the positive database is higher than the HSPs score from the negative database, the test sequence is classified as AMPs. In other words, the classification of the test sequence depended on the class of the training sequence with the maximum HSPs score among all positive and negative training sequences.

However, there are some peptide sequences that have no relationship with any positive or negative training sequences. These sequences have zero hits. The HSPs score of those sequences cannot be obtained if the sequences have zero hits. Therefore, those sequences cannot be predicted by the sequence alignment method. Instead of using the feature selection method [9], the SVM-LZ complexity pairwise algorithm is proposed to predict those remaining unpredictable sequences.

#### 2.2.2. Support Vector Machines- (SVMs-) LZ Complexity Pairwise Algorithm

**Figure 2:**
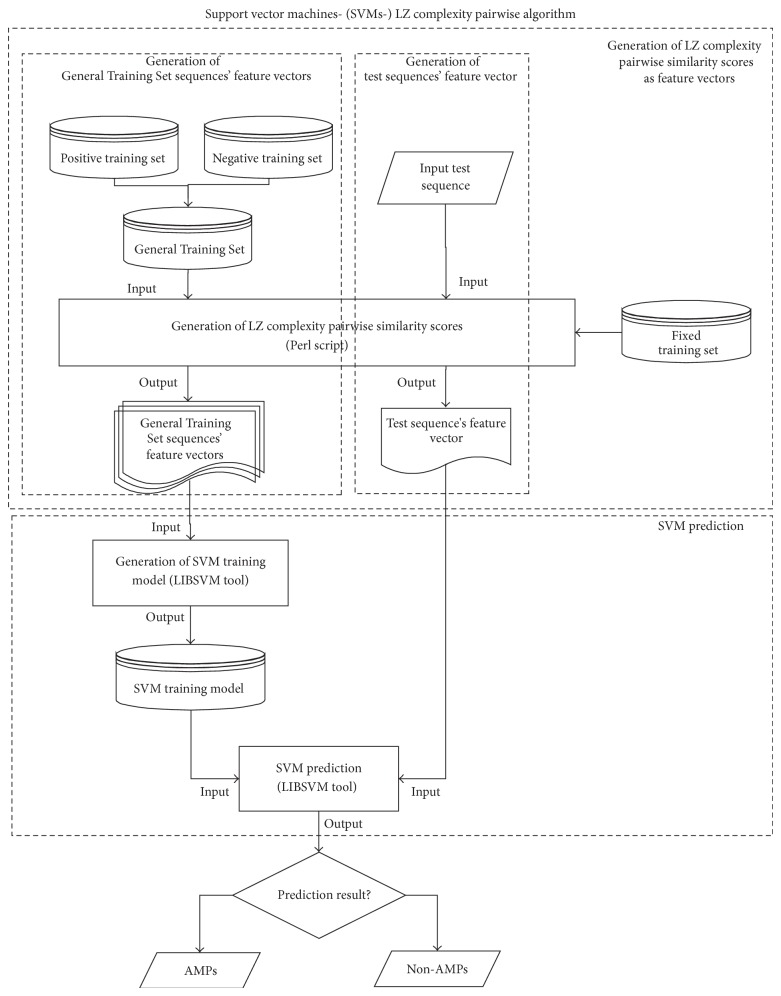
The flowchart of the SVM-LZ complexity pairwise algorithm for stage 2.

Support vector machines- (SVMs-) pairwise algorithm was introduced in [19] with the aim of detecting remote protein evolutionary and structural relationships. This algorithm is the combination of the pairwise sequence similarity algorithm using BLAST and SVM classification. In this paper, a new concept of SVM-LZ complexity pairwise algorithm has been proposed. The SVM-LZ complexity pairwise algorithm is the integration of LZ complexity algorithm [17] and SVM-pairwise algorithm. LZ complexity algorithm is implemented to compute the pairwise similarity scores. Based on LZ complexity pairwise similarity scores, SVM classification is performed to predict AMPs sequences. In this study, the SVM-LZ complexity pairwise algorithm is implemented on those test sequences that cannot be predicted by the sequence alignment method. The flowchart of the SVM-LZ complexity pairwise algorithm is shown in Figure 2. Generally, this algorithm can be categorized into two substages, the generation of LZ complexity pairwise similarity scores as feature vectors and the prediction based on SVM classification.

**Figure 3:**
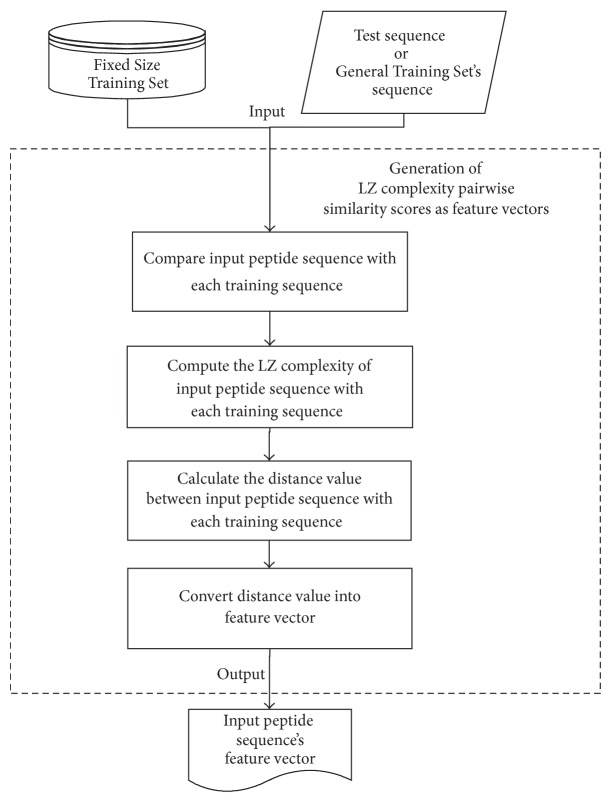
The flowchart of the generation of pairwise similarity scores substage.

*Generation of LZ Complexity Pairwise Similarity Scores as Feature Vectors.* Unlike the pairwise similarity concept proposed in [18, 19], in this study, the generation of pairwise similarity scores is based on the LZ complexity algorithm [17].  Figure 3 shows a flow chart of the generation of pairwise similarity scores substage. A fixed-length vector of real number, known as a feature vector, is generated by comparing the test sequence to a group of training sequences. Due to the requirement of the classifiers [19], the feature vector must be a collection of fixed-length vectors. In order to fulfill these requirements, a Fixed Size Training Set needs to be prepared.

The Fixed Size Training Set must contain a fixed number of training sequences. It is a subset of the downloaded dataset from [9] and consists of an equal number of positive and negative training sequences. These sequences have been used to compare the input test sequence to generate a fixed-length pairwise similarity scores based on the LZ complexity algorithm. In this study, the optimal result was obtained empirically by setting the size of Fixed Size Training Set to 1000 for the normal training set and 500 for the <0.7 training set, which has similarity of less than 70%.

As mentioned before, the generation of feature vector of a peptide sequence is based on the LZ complexity concept [17]. LZ complexity is suitable for calculating the distance between those AMPs sequences because they have a finite number of letters in the sequences. In order to obtain the LZ complexity score of a sequence, the production history of the sequence needs to be identified by parsing the sequence [17]. After the parsing process, the number of components in the history of the sequence can be identified. This number represents an exhaustive history of the sequence. The LZ complexity of a sequence *c*(*S*) is shown in

(1) $$\begin{array}{c} c\left(s\right)=\min\left\{c_{H}\left(S\right)\right\}, \end{array}$$

where *c*(*S*) is the value of LZ complexity of sequence *S*. It is also known as the exhaustive history of sequence *S*. The *c*_*H*_(*S*) is the number of components in the history of a sequence, *S*. Given a test sequence, *X*, and a training sequence, *Y*, the similarity score between sequences *X* and *Y* can be calculated by applying (2), where *c*(*X*), *c*(*Y*), *c*(*XY*), and *c*(*YX*) are the exhaustive histories of sequences *X*, *Y*, *XY*, and *YX*, respectively. The exhaustive history of a sequence is the minimum number of the components in the history of the sequence that can be identified after the parsing process. Consider

(2) $$\begin{array}{c} d\left(X,Y\right)=\frac{\max\left[c\left(XY\right)-c\left(X\right),c\left(YX\right)-c\left(Y\right)\right]}{\max\left[c\left(X\right),c\left(Y\right)\right]}. \end{array}$$

An example of the parsing process to identify the exhaustive history of a sequence and the calculation of the similarity score between two sequences are given below. Given a sequence *X* = *TTCGTA* and a sequence *Y* = *ACTGA*, the exhaustive history of sequence *X* can be identified using the parsing process as follows.

**Step 1 .**

Considering the first letter, *T*,

 since this is the first starting alphabet, *s* and *q* are unknown so
  (3) $$\begin{array}{c} H_{E}\left(X\right)=T•. \end{array}$$

**Step 2 .**

Considering the next letter, *T*,

 
*s* = *T*; *q* = *T*; *sq* = *TT*; and *sqπ* = *T* since *q* ∈ *sqπ* so
  (4) $$\begin{array}{c} H_{E}\left(X\right)=T•T. \end{array}$$

**Step 3 .**

Considering the next letter, *C*,

 
*s* = *TT*; *q* = *TC*; *sq* = *TTC*; and *sqπ* = *TT* since *q* ∉ *sqπ* so
  (5) $$\begin{array}{c} H_{E}\left(X\right)=T•TC. \end{array}$$

**Step 4 .**

Considering the next letter, *G*,

  
*s* = *TTC*; *q* = *G*; *sq* = *TTCG*; and *sqπ* = *TTC* since *q* ∉ *sqπ* so
  (6) $$\begin{array}{c} H_{E}\left(X\right)=T•TC•G. \end{array}$$

**Step 5 .**

Considering the next letter, *T*,

 
*s* = *TTCG*; *q* = *T*; *sq* = *TTCGT*; and *sqπ* = *TTCG* since *q* ∈ *sqπ* so
  (7) $$\begin{array}{c} H_{E}\left(X\right)=T•TC•G•T. \end{array}$$

**Step 6 .**

Considering the last letter, *A*,

 
*s* = *TTCGT*; *q* = *TA*; *sq* = *TTCGTA*; and *sqπ* = *TTCGT* since *q* ∉ *sqπ* so
  (8) $$\begin{array}{c} H_{E}\left(X\right)=T•TC•G•TA. \end{array}$$

The symbol of “•” represents the separation of the components during the parsing process of the LZ complexity method. For example, for an exhaustive history of sequence *X*, *H*_*E*_(*X*) = *T*•*TC*•*G*•*TA*•, there are four symbols of “•” to separate four different components (*T*, *TC*, *G*, and *TA*). Hence the LZ Complexity of *X* is equal to 4, since there are 4 components shown by the exhaustive history of sequence *X*. By applying the same procedure, the exhaustive histories of sequences *Y*, *XY*, and *YX* can be obtained as 5, 7, and 8, respectively. After finding the exhaustive histories of sequences *X*, *Y*, *XY*, and *YX*, a similarity score between sequences *X* and *Y* can be calculated by applying (2). In this example, the similarity score between sequences *X* and *Y* is equal to 0.6.

Once the similarity scores between test sequence and all sequences in the Fixed Size Training Set are obtained, the scores are organized into a feature vector. The size of the feature vector depends on the size of the Fixed Size Training Set.

*SVM Prediction.* A support vector machine (SVM) [18, 19, 22] is often used as a classifier in the Bioinformatics field. In this study, a peptide sequence is represented by feature vector that consists of a list of pairwise similarity scores based on LZ complexity. As stated in Figure 2, SVM is used to perform classification of AMPs sequences by applying LIBSVM tool [22].

**Figure 4:**
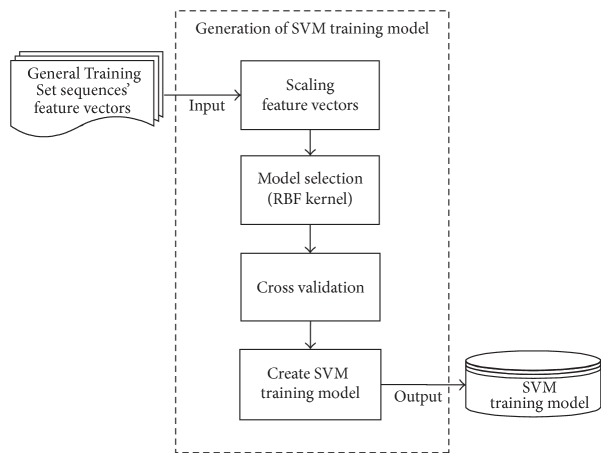
The flowchart of the SVM training model generation.

Before performing prediction on test sequences, a SVM training model is generated.  Figure 4 shows the steps of generation of SVM training model for this study. In order to generate a training model for AMPs prediction, a “General Training Set” has to be prepared. All sequences in the General Training Set are formed by the training sequences that cannot be predicted by the sequence alignment method. This training set consists of an equal number of positive training and negative training sequences. Equation (9) shows the relationship between the size of “General Training Set,” *S*_GT_, and the number of the remaining positive sequences, *S*_RP_:

(9) $$\begin{array}{c} S_{\text{GT}}=2\times \left(S_{\text{RP}}-1\right). \end{array}$$

All the training sequences in General Training Set, which are represented by feature vectors, need to be categorized and labeled with their respective classes. The feature vectors that represent the positive training sequences are labeled as “+1” whereas for the negative training sequences the feature vectors are labeled as “−1.” The feature vectors are scaled into the range of [−1, 1]. Scaling the feature vectors before applying SVM for both test and training sequences is very important as it can improve the accuracy of the AMPs prediction. This is because the scaling process can help to avoid attributes in greater numeric ranges dominating those in smaller ranges. In addition, scaling can also avoid the numerical difficulties during calculation as kernel values usually depend on the inner products of feature vectors.

As stated in [22], the radial basis function (RBF) kernel is suitable to be used to train the SVM model. There are two parameters, *C* and *γ*, that need to be considered when the RBF kernel is used. Therefore, grid search cross validation [22] is carried out in order to obtain the values of *C* and *γ* that can achieve the best accuracy of AMPs prediction. In this study, the optimal performance of the proposed algorithm for normal training set is obtained by setting the parameters *C* and *γ* to 4 and 3.91 × 10^−3^, respectively. Meanwhile, for the <0.7 training set, the optimal value of parameters *C* and *γ* are 16 and 4.88 × 10^−4^, respectively. After attaining the best parameter, the SVM training model is trained by applying the* RBF* kernel with the best parameters. During the prediction of the test sequences, the feature vectors that represented the test sequences also needed to be scaled into the range of [−1,1]. The classification for the scaled test feature vectors is performed based on the trained SVM model.

## 3. Results and Discussion

In this study, the performance of the proposed algorithm was analyzed using both the jackknife test [9, 23] and independent test. In the jackknife test, prediction is done on every training sequence. During the prediction, each peptide sequence is singled out to become the test sequence and the remaining sequences become the training set. As in [9], (10) is used to reflect the prediction quality:

(10) Sn=TPTP+FN,Sp=TNTN+FP,AC⁡=TP+TNTN+TP+FN+FP,MCC=TP∗TN−(FN∗FP) ×TP+FN∗TN+FPkkkk∗TP+FP∗TN+FN−1/2,

where *S*_*n*_ reflects the sensitivity, *S*_*p*_ is the specificity, AC stands for the accuracy, and MCC represents the Mathews correlation coefficient. Besides that, TP (true positive) represents correct predictions of the positive dataset; FP (false positive) represents incorrect predictions of the negative dataset; TN (true negative) represents correct predictions of the negative dataset; and FN (false negative) represents incorrect predictions of the positive dataset. Applying (10), the performance of the proposed algorithm can be measured and compared to previously proposed methods.

Besides the jackknife test, an independent test has also been used to evaluate the performance of the proposed algorithm. The independent test is used to demonstrate the performance of a predictor for practical application [24]. The sequences in the test sets were used to evaluate the performance of the proposed algorithm.

In the experimental setup stage, the proposed algorithm was performed in Microsoft Windows Operation System using a modern notebook (Intel i7-2670QM CPU @ 2.20 GHz, 8 GB RAM). The main software language used to build the predictor was Perl. All the Perl coding was performed using Active Perl version 5.16.1 with command prompt windows. To perform the sequence alignment method, BLASTP [21] was used to calculate the HSPs scores using default parameter. The version of BLASTP was 2.2.23. The Perl and LIBSVM [22] were used to perform the SVM-LZ complexity pairwise algorithm. The pairwise similarity scores were generated using Perl (the script is in Supplementary Material available online at http://dx.doi.org/10.1155/2015/212715) based on the LZ complexity [17]. The LIBSVM was used to perform SVM modeling and prediction based on the feature vectors that were generated by Perl. The version of LIBSVM was 3.14.

In this study, six experiments were conducted to analyze the performance of the proposed algorithm. First, an experiment was carried out to study the effect of pairwise similarity concept and SVM as the classifier on our proposed algorithm. Second, another experiment was executed to observe the differences between the sequence alignments with different version of BLASTP. As the version of BLASTP used in [9] was unknown, this experiment was conducted to compare the performance of different versions of BLASTP to obtain the optimal result. Third, an experiment was designed to study the effectiveness of using SVM pairwise to replace the feature selection method. The jackknife tests in the first three experiments were performed using <0.7 training set in order to compare with the results stated in [9]. To investigate the effect of sequence homology on the performance of the methods, the normal training set and the <0.7 training set were used to perform both jackknife test and independent test in the fourth experiment. Besides, in this experiment, the performance of sequence alignment and our SVM-LZ complexity pairwise algorithm was compared, and the Wang test set was used in the independent test. In order to compare the performance between the proposed algorithm with the previously proposed methods, an independent test on normal training set was carried out using the Wang test set in the fifth experiment. Lastly, to confirm the effectiveness of the proposed method in the prediction of the newly identified AMPs, an independent test was performed using the CAMP test set [20].

*Comparison of the Effectiveness of LZ Complexity Pairwise Similarity Concept and SVM as Classifier with Other Methods and Classifiers.* In the complexity-based distance measure [25], a test sequence is represented by a single distance score that is calculated by LZ complexity. For the proposed algorithm, the LZ complexity was implemented together with the pairwise concept. The pairwise similarity concept was implemented to consider the relationship of the test sequence and the training sequences. The test sequence in this project is represented by a feature vector that consists of a list of similarity scores between test and training sequences.

As we can see from Table 1, the *S*_*n*_ for the NNA- (nearest neighbor algorithm-) LZ complexity pairwise and SVM-LZ complexity pairwise are better than the complexity-based distance measure in the jackknife test when using <0.7 training set. This demonstrates that the pairwise concept has a significant improvement in the sensitivity measure. It is believed that the sensitivity measure is an important parameter for AMPs prediction as the sensitivity measure represents the total number of AMPs sequences that can be predicted correctly by the AMPs predictor. Using a high sensitivity AMPs predictor, the number of correctly extracted AMPs sequences can be increased. Therefore, the missing probability of extracting the real AMPs from unknown sequences could be reduced accordingly.

As we can see from Tables 1 and 2, the SVM-LZ complexity pairwise algorithm depicted a higher overall success rate compared with the nearest neighbor algorithm- (NNA-) LZ complexity pairwise. This proves that SVM is a better and more stable classifier than NNA for AMPs prediction.

*Comparison of the Sequence Alignment Method with Different Version of BLASTP.* The sequence alignment method in the proposed algorithm was performed using BLASTP [21]. However, in this study, it is noticed that the HSPs scores for the same sequence varied if different versions of BLASTP were used. Because the version of BLASTP used by Wang and colleagues was not specified in [9], the BLASTP version 2.2.23 was used in this study. In this study, jackknife test was used to measure the performance of different version of BLASTP. As stated in [9], the jackknife test for sequence alignment was performed using dataset that has less than 70% similarity. To make the comparison more meaningful, the <0.7 training set was used to perform this experiment. The prediction results are shown in Table 3 together with the results of the sequence alignment method performed by Wang and colleagues. Due to a different version of BLASTP, the simulation results obtained were different from [9]. The sequence alignment method cannot deal with all sequences because there was no hit found between the test and training sequences. With a total of 9731 training sequences, only 7158 training sequences were predicted, as shown in Table 3. The values for *S*_*n*_, *S*_*p*_, AC, and MCC were 92.22%, 79.19%, 80.46%, and 0.4720, respectively.

*Comparison of SVM-LZ Complexity Pairwise with Feature Selection Method*. Due to the limitation of the sequence alignment method, the feature selection method was introduced in [9] to predict the remaining sequences. In this study, the SVM-LZ complexity pairwise algorithm was used to replace the feature selection method in order to obtain a better performance. The remaining unpredictable test sequences were predicted using the SVM-LZ complexity pairwise algorithm. These sequences were represented by feature vectors. Each feature vector was a list of fixed length similarity scores between the test and training sequences. The similarity score was generated based on the LZ complexity concept by comparing the test sequence to one of the training sequences. The test sequence's feature vector was used to perform SVM classification. As shown in Table 4, the SVM-LZ complexity pairwise algorithm was proven to have a better *S*_*n*_ value as compared to the feature selection method.

**Figure 5:**
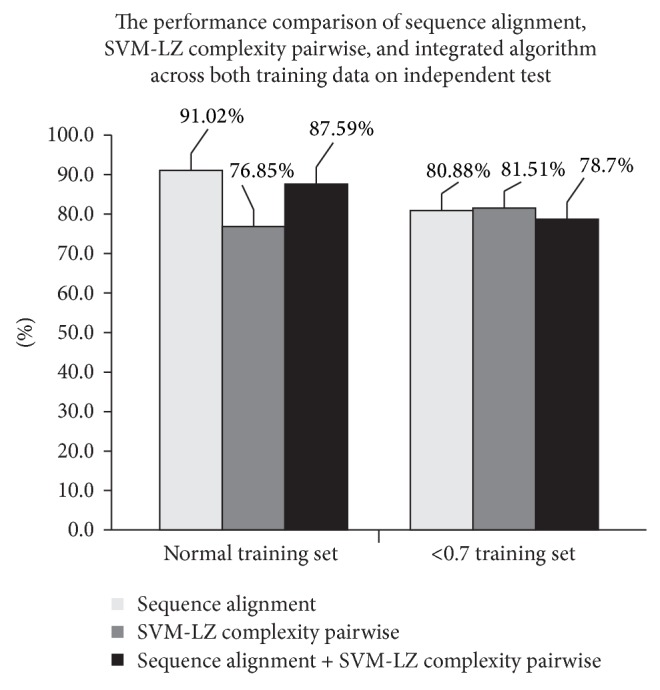
The performance comparison of sequence alignment, SVM-LZ complexity pairwise, and integrated algorithm across both training data on independent test using Wang test set.

*Comparison of the Performance of Sequence Alignment, SVM-LZ Complexity Pairwise, and Proposed Algorithm.* In the proposed integrated method, both sequence alignment and SVM-LZ complexity pairwise have been used to predict peptide sequence. Besides comparing with the previously proposed methods, the performance of each standalone method proposed in this study has been evaluated using independent test for both training sets. As shown in Table 5 and Figure 5, the sequence alignment method achieved the highest sensitivity value in the normal training set. However, as mentioned earlier, the sequence alignment method was unable to predict all peptide sequences because HSPs score cannot be generated by the BLASTP. On the other hand, although the performance of the SVM-LZ complexity pairwise is not as good as the sequence alignment method, but it can predict all peptide sequences. Hence, by combining both techniques, the limitation of the sequence alignment method can be solved. As for the <0.7 training set, although the SVM-LZ complexity pairwise achieved the highest sensitivity value, it is still lower than the integrated method that used the normal training set. Hence, in this paper, we propose to use the integrated method to predict the peptide sequences. As stated in [9], in order to achieve the highest accuracy, it is better to use all training data.

*Comparison of the Proposed Algorithm with the Previously Proposed Methods.* By combining the results of the sequence alignment and the SVM-LZ complexity pairwise algorithm, the overall success rate for this integrated method is shown in Table 6. As presented in Table 6, the jackknife test performed using normal training set has a very high overall success rates. The values for *S*_*n*_, *S*_*p*_, AC, and MCC were 95.28%, 87.25%, 88.98%, and 0.736, respectively. When <0.7 training set was used, the values for *S*_*n*_, *S*_*p*_, AC, and MCC were 88.74%, 79.17%, 80.02%, and 0.437, respectively. As per Table 6, the sensitivity of the proposed algorithm in this project is 8% higher than the integrated method proposed by Wang et al. [9] for jackknife test using <0.7 training set. Therefore, the optimal performance of AMPs predictor can be obtained by combining the sequence alignment and SVM-LZ complexity pairwise algorithm.

**Figure 6:**
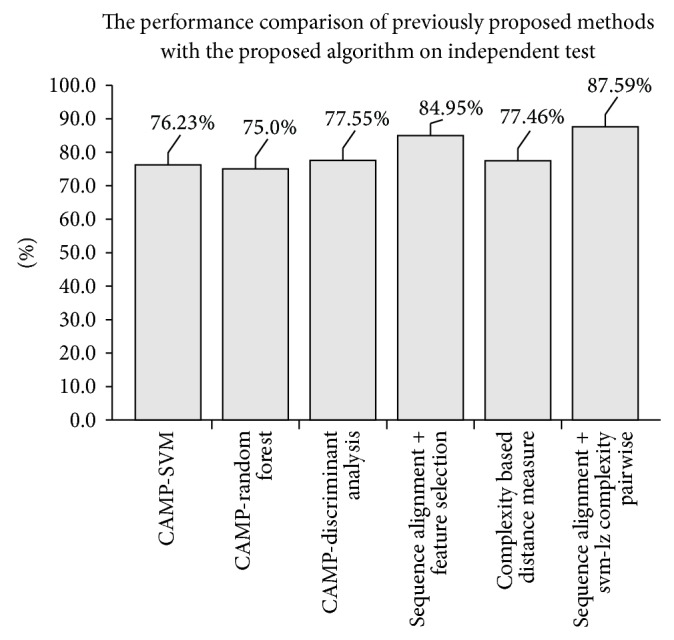
The performance comparison of previously proposed methods with the proposed algorithm on independent test using Wang test set.

Besides the jackknife test, the independent test was also used to evaluate the performance of the proposed algorithm. An independent test is used for demonstrating the performance of a predictor for practical application [24].  Table 7 and Figure 6 show the results of the independent test for the proposed algorithm and previously proposed methods from CAMP and Wang et al. [9]. As stated in [9], the normal training set is used to perform the independent test because all training data must be used in order to have a better performance upon testing. Per Table 7, the proposed algorithm in this project had the highest sensitivity at 87.59%. This results indicate that the proposed algorithm in this project is suitable to be used as an AMPs predictor.

**Figure 7:**
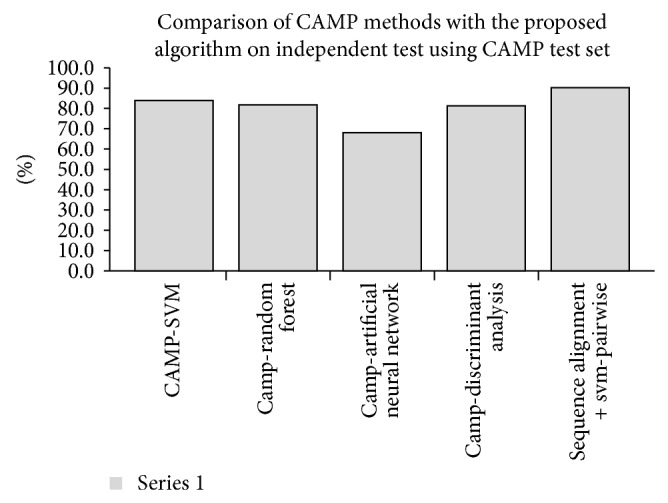
The performance comparison of CAMP methods with the proposed algorithm on independent test using CAMP test set.

*Comparison of the Proposed Algorithm with the Previously Proposed Methods Using CAMP Test Set.* As mentioned earlier, recently CAMP database has a major update [20]. To confirm the effectiveness of the proposed algorithm in the prediction of the newly identified AMPs, it was evaluated and compared with the methods proposed in [20] using the CAMP test set. As we can see from Table 8 and Figure 7, our proposed algorithm achieved the highest sensitivity at 90.25%. The experimental results show that the proposed algorithm consistently outperforms the methods proposed in [20].

## 4. Discussion

It has been reported that the diversity of the amino acid sequence, structure, and biological activity of AMPs are high. This is because the AMP genes are evolved for the survival of the organisms in different microbial environment [26]. Hence, a single threshold of similarity might not be effective to predict the antimicrobial peptides. In this aspect, our proposed method considers a profile of pairwise similarities to both AMPs and non-AMPs sequences. The generated feature vectors, which consist of pairwise LZ-complexity scores, amplify the similarities and differences between the antimicrobial and nonantimicrobial peptides. As we can see from the experimental results, the concept of pairwise similarity scores helps to increase the prediction accuracy. Our findings are consistent with what had been reported in [18], where they also utilize the concept of pairwise similarity scores to improve the prediction accuracy of allergen from the primary sequence of protein.

## 5. Conclusion

In this study, the proposed algorithm was the combination of the sequence alignment method and SVM-LZ complexity pairwise algorithm.

The sequence alignment method was developed using BLASTP to calculate the HSPs scores between test and training sequences. The classification of the test sequence depended on the class of the training sequence that has the highest HSPs score. However, the sequence alignment was unable to deal with all sequences as there were some sequences which had no relationship with the training sequences. Thus, the SVM-LZ complexity pairwise algorithm was newly introduced to predict the remaining sequences. In the SVM-LZ complexity pairwise algorithm, the peptide sequences were represented by the fixed length feature vectors. These feature vectors consisted of a list of pairwise similarity scores. The LIBSVM tool was used to perform SVM classification on the test sequence based on the feature vector. As we can see from the experimental results, the proposed algorithm was proven to have the best performance on both jackknife and independent tests based on sensitivity. The proposed algorithm obtained a sensitivity of 95.28% in the jackknife test and 87.59% in the independent test for normal training set. When <0.7 training set was used, the sensitivity obtained for the jackknife test and that for the independent test are 88.74% and 78.70%, respectively.

By applying the proposed algorithm, it is believed that the researchers were able to effectively predict AMPs from unknown protein peptide sequences with higher sensitivity. These AMPs predictors are believed to be able to help scientists or researchers to understand the task of peptides in antimicrobial activity, identify the natural AMPs, and develop and design new synthetic AMPs to replace currently available antibiotics in order to fight against microorganisms.

For the future works, the proposed method can be further improved by replacing the support vector machines (SVMs) classifier with sparse representations classifier (SRC) [27–29] or fuzzy k-nearest neighbour method [30]. Besides, we also believe that, by implementing the manifold fitting approach proposed by Zhang et al. [31], the performance of AMPs predictor can be enhanced. In this study, the proposed method is used to predict AMPs sequences from unknown peptide sequences. In the near future, the concept of this new proposed integrated method can be implemented to classify AMPs sequences based on the biosynthetic machine, biosynthetic sources, biosynthetic functions, or molecular properties or molecular targets [32].

## Supplementary Material

The supplementary material includes the Perl script to generate the pairwise similarity scores based on LZ-complexity. The details explanation on how to use the script are written inside the file.

## Figures and Tables

**Table 1:** The effect of pairwise similarity concept and SVM based on the Jackknife test.

Method/algorithm	*S*_*n*_	*S*_*p*_	AC	MCC
Complexity-based distance measure	64.94%	95.16%	92.46%	0.5663
NNA-LZ complexity pairwise	76.60%	74.52%	74.63%	0.2537
SVM-LZ complexity pairwise	85.00%	86.47%	86.37%	0.4624

**Table 2:** The effect of pairwise similarity concept and SVM based on the independent test.

Method/algorithm	Number of predicted sequences	Number of correctly predicted sequences	*S*_*n*_
Complexity-based distance measure	1136	880	77.46%
NNA-LZ complexity pairwise	1136	0	0%
SVM-LZ complexity pairwise	1136	870	76.58%

**Table 3:** The performance comparison between both sequence alignment methods on jackknife test.

Method	Number of predicted sequences	*S*_*n*_	*S*_*p*_	AC	MCC
Sequence alignment [9]	5855	91.22%	95.55%	95.12%	0.7723
Sequence alignment by BLAST ver. 2.2.23	7158	92.22%	79.19%	80.46%	0.4720

**Table 4:** The performance comparison between the feature selection method and SVM-LZ complexity pairwise algorithm on jackknife test.

Method/algorithm	Number of predicted sequences	*S*_*n*_	*S*_*p*_	AC	MCC
Feature selection [9]	3876	56.83%	93.19%	90.58%	0.6426
SVM-pairwise	2573	75.00%	79.10%	78.82%	0.3171

**Table 5:** The performance comparison of sequence alignment, SVM-LZ complexity pairwise, and our integrated algorithm across both training data on independent test using Wang test set.

Type of training data	Algorithm	Number of predicted sequences	Number of correctly predicted sequences	*S*_*n*_
Normal training set	Sequence alignment	1025	933	91.02%
	SVM-pairwise	All (1136)	870	76.85%
	Sequence alignment + SVM-pairwise	All (1136)	995	87.59%

<0.7 training set	Sequence alignment	1004	812	80.88%
	SVM-pairwise	All (1136)	926	81.51%
	Sequence alignment + SVM-pairwise	All (1136)	894	78.70%

**Table 6:** The performance comparison of both integrated algorithms on jackknife test.

Algorithm	Type of training set	Number of predicted sequences	*S*_*n*_	*S*_*p*_	AC	MCC
Sequence alignment + SVM-LZ complexity pairwise	Normal	12766	95.28%	87.25%	88.98%	0.736
	<0.7	9731	88.74%	79.17%	80.02%	0.437
Sequence alignment + feature selection [9]	<0.7	9731	80.23 %	94.59 %	93.31 %	0.7312

**Table 7:** The performance comparison of previously proposed methods with the proposed algorithm on independent test using Wang test set.

Method/algorithm	Number of predicted sequences	Number of correctly predicted sequences	*S*_*n*_
CAMP-SVM [9]	1136	866	76.23%
CAMP-random forest [9]	1136	852	75.00%
CAMP-discriminant analysis [9]	1136	881	77.55%
Sequence alignment + feature selection [9]	1136	965	84.95%
Complexity based distance measure	1136	880	77.46%
Sequence alignment + SVM-pairwise	1136	995	87.59%

**Table 8:** The performance comparison of CAMP methods with the proposed algorithm on independent test using CAMP test set.

Method/algorithm	Number of predicted sequences	Number of correctly predicted sequences	*S*_*n*_
CAMP-SVM [20]	2420	2030	83.88%
CAMP-random forest [20]	2420	1978	81.74%
CAMP-artificial neural network [20]	2420	1648	68.10%
CAMP-discriminant analysis [20]	2420	1967	81.28%
Sequence alignment + SVM-pairwise	2420	2184	90.25%
